# Detection of Fuchs’ Uveitis Syndrome From Slit-Lamp Images Using Deep Convolutional Neural Networks in a Chinese Population

**DOI:** 10.3389/fcell.2021.684522

**Published:** 2021-06-18

**Authors:** Wanyun Zhang, Zhijun Chen, Han Zhang, Guannan Su, Rui Chang, Lin Chen, Ying Zhu, Qingfeng Cao, Chunjiang Zhou, Yao Wang, Peizeng Yang

**Affiliations:** ^1^The First Affiliated Hospital of Chongqing Medical University, Chongqing Key Laboratory of Ophthalmology and Chongqing Eye Institute, Chongqing Branch of National Clinical Research Center for Ocular Diseases, Chongqing, China; ^2^School of Computer Science and Technology, Harbin Institute of Technology, Harbin, China

**Keywords:** Fuchs’ uveitis syndrome, diffuse iris depigmentation, slit-lamp images, deep convolutional neural model, deep learning

## Abstract

Fuchs’ uveitis syndrome (FUS) is one of the most under- or misdiagnosed uveitis entities. Many undiagnosed FUS patients are unnecessarily overtreated with anti-inflammatory drugs, which may lead to serious complications. To offer assistance for ophthalmologists in the screening and diagnosis of FUS, we developed seven deep convolutional neural networks (DCNNs) to detect FUS using slit-lamp images. We also proposed a new optimized model with a mixed “attention” module to improve test accuracy. In the same independent set, we compared the performance between these DCNNs and ophthalmologists in detecting FUS. Seven different network models, including Xception, Resnet50, SE-Resnet50, ResNext50, SE-ResNext50, ST-ResNext50, and SET-ResNext50, were used to predict FUS automatically with the area under the receiver operating characteristic curves (AUCs) that ranged from 0.951 to 0.977. Our proposed SET-ResNext50 model (accuracy = 0.930; Precision = 0.918; Recall = 0.923; F1 measure = 0.920) with an AUC of 0.977 consistently outperformed the other networks and outperformed general ophthalmologists by a large margin. Heat-map visualizations of the SET-ResNext50 were provided to identify the target areas in the slit-lamp images. In conclusion, we confirmed that a trained classification method based on DCNNs achieved high effectiveness in distinguishing FUS from other forms of anterior uveitis. The performance of the DCNNs was better than that of general ophthalmologists and could be of value in the diagnosis of FUS.

## Introduction

Fuchs’ uveitis syndrome (FUS) is a chronic, mostly unilateral, non-granulomatous anterior uveitis, accounting for 1–20% of all cases of uveitis at referral centers, and is the second most common form of non-infectious uveitis (Yang et al., 2006; Kazokoglu et al., 2008; Abano et al., 2017). It is reported to be one of the most under- or misdiagnosed uveitis entities, with its diagnosis often delayed for years (Norrsell and Sjödell, 2008; Tappeiner et al., 2015; Sun and Ji, 2020). Patients with FUS generally present with an asymptomatic mild inflammation of the anterior segment of the eye (Sun and Ji, 2020). The syndrome is featured by characteristic keratic precipitates (KPs), depigmentation in the iris with or without heterochromia, and absence of posterior synechiae (Tandon et al., 2012). Heterochromia is a striking feature of FUS in white people (Bonfioli et al., 2005). However, iris depigmentation may be absent or subtle, especially in patients from Asian or African populations, who have a higher melanin density in their iris (Tabbut et al., 1988; Arellanes-García et al., 2002; Yang et al., 2006; Tugal-Tutkun et al., 2009). In a previous study on Chinese FUS patients, we described the presence of varying degrees of diffuse iris depigmentation without posterior synechiae rather than heterochromia (Yang et al., 2006). Degrees of diffuse iris depigmentation may be considered as the most sensitive and reliable signs of FUS in Chinese as well as in other highly pigmented populations (Mohamed and Zamir, 2005; Yang et al., 2006). The subtle iris depigmentation is however often neglected, leading to a misdiagnosis (Tappeiner et al., 2015). Many undiagnosed FUS patients are unnecessarily treated chronically or intermittently with topical or systemic corticosteroids or even other immunosuppressive agents, which may lead to cataract formation and severe glaucoma (Menezo and Lightman, 2005; Accorinti et al., 2016; Touhami et al., 2019). Until now, the diagnosis is highly dependent on the skills of the uveitis specialist with broad experience in the detection of subtle iris pigmentation abnormalities in a patient with mild anterior uveitis.

Deep learning (DL), one of the most promising artificial intelligence technologies, has been demonstrated to learn from and make predictions on data sets (He et al., 2019). Deep convolutional neural network (DCNN), a subtype of DL, has proven to be a useful method in image-centric specialties, especially in ophthalmology (Hogarty et al., 2019). The capability of DCNN to learn a complicated representation of the data makes it useful for solving the classification problem to facilitate accurate diagnosis of various diseases (Kapoor et al., 2019; Ting et al., 2019). To offer assistance for ophthalmologists in the screening and diagnosis of FUS, we decided to develop DCNNs to classify slit-lamp images automatically and in this report we show its feasibility in the detection of FUS.

## Materials and Methods

### Data Sets

We designed a retrospective study based on the slit-lamp images of 478 Fuchs patients and 474 non-FUS controls from the uveitis center of the First Affiliated Hospital of Chongqing Medical University during January 2015 to October 2020. The diagnosis of FUS was made according to the criteria by La Hey et al. (1991) in combination with the description for Chinese FUS patients in a previous report from our group (Yang et al., 2006). Non-FUS patients with other uveitis entities (Table 1), who presented with signs and symptoms of anterior uveitis and had images comparable with those in FUS patients, served as controls. All enrolled patients were diagnosed by more than two specialists from referring hospitals and then verified by uveitis specialists from our center. The slit-lamp images of each patient were collected using a digital slit-lamp microscope (Photo-Slit Lamp BX 900; Haag-Streit, Koeniz, Switzerland). These images were taken with a 30° angle using direct illumination and focused on the iris with varying degrees of magnification (10, 16, or 25). To highlight the diffusion and uniformity of the iris depigmentation, only images that covered about half of the iris appearance were included.

**TABLE 1:** Uveitis entities in the Non-Fuchs’ uveitis syndrome group.

Entity	Total	Number (%)	
		The training and validation set	The test set
Idiopathic chronic anterior uveitis	124	100 (26.2)	24 (26.1)
Posner–Schlossman syndrome	83	66 (17.3)	17 (18.5)
Presumed viral anterior uveitis	74	60 (15.7)	14 (15.2)
Acute anterior uveitis	66	53 (13.9)	13 (14.1)
Sarcoidosis	62	51 (13.3)	11 (12.0)
Vogt–Koyanagi–Harada disease	35	28 (7.3)	7 (7.6)
Behcet’s disease	30	24 (6.3)	6 (6.5)
Total	474	382	92

A total of 2,000 standard slit-lamp images were collected anonymously and removing all personal data except types of disease. These images were used as the basis for training DCNNs consisting of 872 slit-lamp images of affected eyes showing the diffuse and uniform iris depigmentation without posterior synechiae from FUS patients (FUS group) and 1,128 images of control eyes from non-FUS patients (non-FUS group). Then, the 20% aggregate images were set as an independent test set to evaluate the effectiveness and generalization ability of DCNNs. The remaining 80% images were randomly and respectively assigned to the training set and the validation set in an 8:2 ratio. The training set was used to train DCNNs, whereas the validation set was utilized to optimize learnable weights and parameters of DCNNs. The images collected from the same patient (left and right eyes or from multiple sessions) could ensure to be not separated between the test set and the other two sets. The study was approved by the Ethical Committee of First Affiliated Hospital of Chongqing Medical University (No. 2019356) and was conducted in accordance with the Declaration of Helsinki for research involving human subjects.

### Development of the DL Algorithm

The slit-lamp images were initially preprocessed to derive data for developing the DCNNs. Each image was resized to 224 × 224 pixels to be compatible with the original dimensions of the experiment networks. Then, the pixel values were scaled to range from 0 to 1. To increase the diversity of the data set and reduce the risk of overfitting, we applied several augmentations to each image, involving random cropped, random rotation, random brightness change, and random flips. Data augmentation is an essential approach to automatically generate new annotated training samples and improve the generalization of DL models (Tran et al., 2017). We obtained samples with shearing with ranges of [−15%, + 15%] of the image width, with rotation [0°, 360°], with brightness change with ranges of [−10%, + 10%], and with or without flipping, thereby generating 10 images per photograph.

Resnet, as a residual deep neural network, was widely used because it is easy to optimize and can gain accuracy from significantly increased depths (He et al., 2016). There are various depths of Resnet structures (Resnet50, Resnet101, and Resnet152), and in this study we used Resnet50 as the experimental models. ResNext, as a new network derived on the basis of Resnet, was included since it can improve accuracy while maintaining Resnet’s high-portability benefits (Xie et al., 2017). Moreover, we introduced a new “attention” unit: the Squeeze-and-Excitation (SE) module. This module allows the network to selectively emphasize informative features and suppresses less useful ones (Hu et al., 2017). After uniting data with the SE module, four different networks (Resnet50, SE-Resnet50, ResNext50, and SE-ResNext50) were included. For comparison, we also selected the Xception network, containing a new convolutional structure named depth-wise separable convolutions that used less parameters and were defined or modified easily (Chollet, 2017). These five classical DCNNs were pre-trained to running the detection of FUS.

**FIGURE 1:**
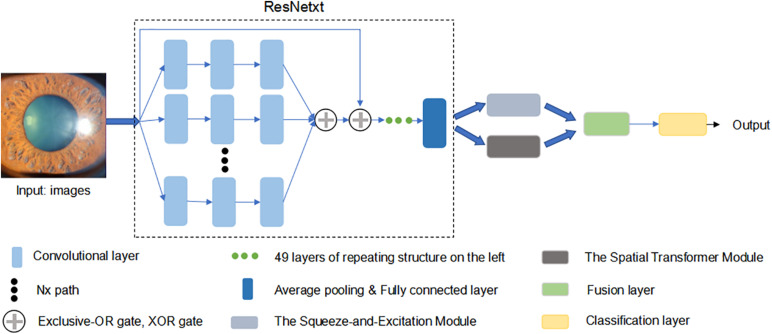
The architecture of SET-ResNext50. We used ResNext50 as backend uniting a mixed “attention” module (the Squeeze-and-Excitation module and the Spatial Transform module). This network was pre-trained in a classification dataset Imagenet to initialize its parameters. Then, we modified the last layer to output a two-dimension vector and updated all the parameters by using the cross entropy.

To improve test accuracy, we constructed a new optimized model, using ResNext50 as the backend. Considering the ResNext50 lack of ability to be spatially invariant to the input data, we introduced a “Spatial Transformer (ST)” module, which is another “attention” unit to provide explicit spatial transformation capabilities. This module performs the ability to learn invariance to translation, scale, rotation, and more generic warping, resulting in state-of-the-art performance (Jaderberg et al., 2015). Applying a mixed “attention” module (SE and ST module), our new model (SET-ResNext50) could not only learn the informative features but also focus on the informative location. We also conducted ablation experiments (ST-ResNext50: ResNext50 with ST module) to verify the effectiveness of our proposed mixed “attention” module. Moreover, we manually tuned various combinations of the hyper-parameters to ensure that the trained models met our experimental requirements. The architecture of SET-ResNext50 is shown in Figure 1.

We developed DCNNs to classify the slit-lamp images into two categories: FUS and Non-FUS. To optimize the models and achieve a better training effect, each model was pre-trained in a classification dataset Imagenet (Deng et al., 2009) to initialize its parameters. Then, we used a 2080 Ti GPU and mini-batches of 96 inputs. The cross entropy was used as a loss function to update all the parameters of the network. The Adam optimizer was used as an optimization function with a learning rate of 10^–4^. The last layer of DCNNs was modified to output a two-dimension vector. We applied fivefold cross-validation for each DCNN to test the statistical significance of the developed models. The heat maps highlighted lesions and showed the location on which the decision of the algorithm was based (Zhou et al., 2016).

### Evaluation of the DL Algorithm

The performance of our experimental models was evaluated in an independent test data set. Images obtained from the same patient could ensure to be not split across the test set and the other two sets. The fivefold cross-validation binary classification results of each model were used to calculate the mean and standard deviation for testing the statistical significance of the developed models. We used receiver operating characteristic (ROC) curves, with calculations of an area under the receiver operating characteristic curves (AUCs), as an index of the performance of our automated models (Carter et al., 2016). AUCs were computed for each finding with 95% confidence intervals computed by the exact Clopper–Pearson method using the Python scikit-learn package version 0.18.2. Precision, accuracy, and recall were used to evaluate the FUS classification performance of our developed models. To make a trade-off between precision and accuracy, F1 measures were added to assess the effectiveness. SPSS version 24.0 (IBM) was used to compare quantitative variables by Student’s *t*-test.

### Comparison of the Networks With Human Ophthalmologists

We compared the performance between seven DCNNs and the clinical diagnosis of ophthalmologists. We chose six ophthalmologists in two different levels (attending ophthalmologists with at least 5 years of clinical training in uveitis from our center: Dr. Zi Ye, Shenglan Yi, and Handan Tan; resident ophthalmologists with 1–3 years of clinical training in ophthalmology from other eye institutes: Dr. Jun Zhang, Yunyun Zhu, and Liang Chen). None of them has participated in the current research. The slit-lamp images were subjected to each ophthalmologist alone and were requested to assign one of three labels to each image, i.e., FUS, uncertain, non-FUS. They were strongly advised not to choose the uncertain label because it is considered as a wrong answer for final evaluation.

## Results

### Baseline Characteristics

A total of 2,000 slit-lamp images from 478 FUS patients and 474 non-FUS controls were collected and assessed during the study period. The non-FUS group included various forms of anterior uveitis and panuveitis with a presentation of anterior uveitis. The types and proportion of non-FUS cases are listed in Table 1. The 2,000 images were assigned to the training set, the validation set, and the test set. The training and validation set (1,600 images) included 698 images from 380 FUS patients and 902 images from 382 non-FUS patients, and the test set (400 images) consisted of 174 images from 98 FUS patients and 226 images from 92 non-FUS patients.

### Performance of the DL Algorithm

After applying fivefold cross-validation, we calculated the mean value and standard deviation to evaluate the performance of our developed models. Performance results are reported in Table 2. In aggregate, the performance of all trained models showed promising outcomes when considering the selected metrics including accuracy, precision, F1 measure, and recall. In the test set of 400 images, seven DCNNs achieved the accuracy of 0.883–0.930, while F1 measures were 0.866–0.920. We found that the performance of ResNext50 was better than that of SE-ResNext50 or ST-ResNext50, demonstrating that the combination of SE or ST modules with the model would not improve the effectiveness of our networks. However, after uniting with the mixed “attention” module, our SET-ResNext50 model consistently outperformed other network models with its performance (accuracy = 0.930; Precision = 0.918; Recall = 0.923; F1 = 0.920). There were significant differences in accuracy between SET-ResNext50 and the other models except ResNext50 (*p* < 0.05). The F1 measure of SET-ResNext50 was higher than that of ResNext50 (*p* = 0.043), which showed that SET-ResNext50 is more superior than other models.

**TABLE 2:** Performance of the deep convolutional neural networks with fivefold cross-validation and the compared methods in the test set.

		Accuracy (SD)	Precision (SD)	Recall (SD)	F1-measure (SD)	*P*-value*
The classical DCNNs	Xception	0.883 (0.007)	0.861 (0.039)	0.875 (0.047)	0.866 (0.008)	<0.01
	Resnet50	0.903 (0.016)	0.879 (0.047)	0.905 (0.044)	0.890 (0.016)	0.044
	SE-Resnet50	0.893 (0.025)	0.855 (0.040)	0.909 (0.052)	0.880 (0.028)	0.007
	ResNext50	0.904 (0.015)	0.889 (0.019)	0.890 (0.048)	0.889 (0.020)	0.052
	SE-ResNext50	0.893 (0.024)	0.897 (0.038)	0.852 (0.045)	0.873 (0.029)	<0.01
Ablation experiments	ST-ResNext50	0.896 (0.013)	0.885 (0.036)	0.879 (0.068)	0.880 (0.021)	0.014
Our proposed model	SET-ResNext50	0.930 (0.005)	0.918 (0.028)	0.923 (0.027)	0.920 (0.004)	−
Ophthalmologists	Resident	0.597 (0.045)	0.539 (0.054)	0.638 (0.095)	0.578 (0.009)	<0.01
	Attending	0.709 (0.032)	0.648 (0.018)	0.722 (0.100)	0.681 (0.056)	<0.01

*Comparison of accuracy with the SET-ResNext50.

*SD, standard deviation; SE, Squeeze-and-Excitation; ST, Spatial Transformer.*

**FIGURE 2:**
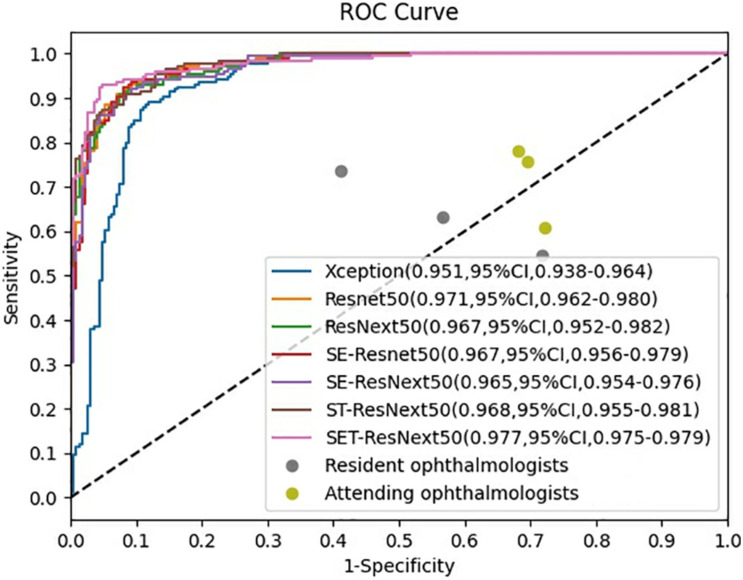
Receiver operating characteristic curves of the performance for diagnosis of Fuchs uveitis syndrome in the test set. SET-ResNext50 achieved an AUC of 0.977 (95%CI, 0.975–0.979), which outperformed other developed networks and outperformed all the ophthalmologists by a large margin.

The ROCs and AUCs are reported in Figure 2. AUCs were 0.951–0.977 of DCNNs, which also demonstrate good performance of the developed models. SET-ResNext50 with its AUC of 0.977 showed that this model could be the optimal choice to facilitate the diagnosis of FUS among seven networks. The other metrics in Table 2 also echoed these observations.

**FIGURE 3:**
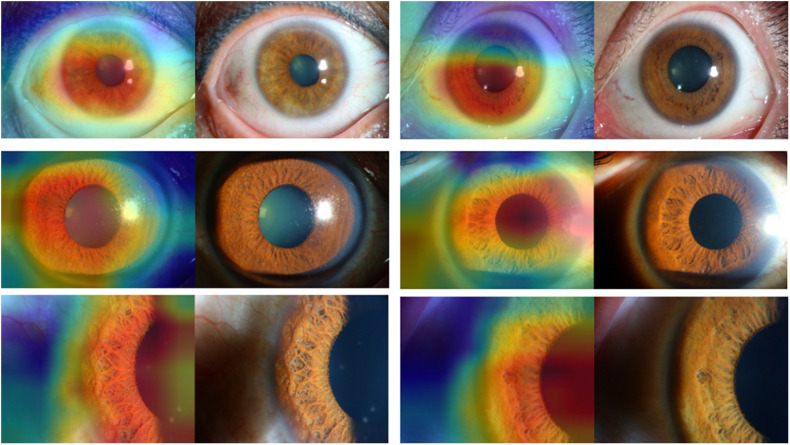
The heat maps of the SET-ResNext50 model in slit-lamp image with Fuchs uveitis syndrome demonstrating representative findings, shown in the original slit-lamp image (right) and corresponding heat map for target areas (left).

**FIGURE 4:**
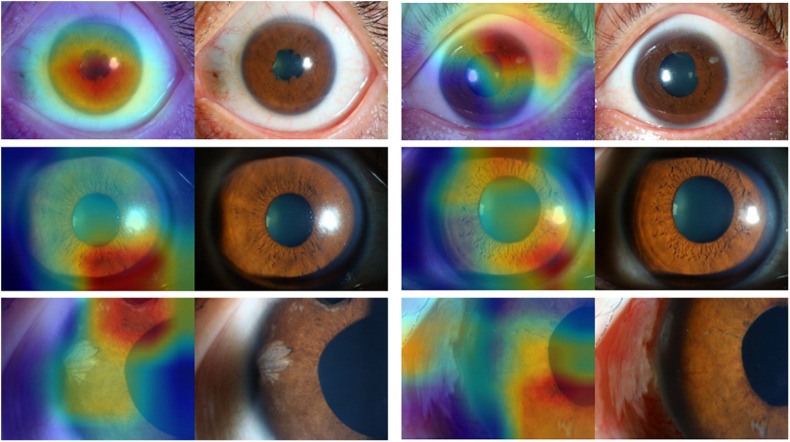
The heat maps of the SET-ResNext50 model in slit-lamp image with non-Fuchs uveitis syndrome demonstrating representative findings, shown in the original slit-lamp image (right) and corresponding heat map for target areas (left).

Figures 3, 4 present the examples of heat maps of SET-ResNext50 model for each finding, accompanied by the corresponding original image. The heat maps showed the most apparently affected region in slit-lamp images. This region was the most important indicator to distinguish FUS from non-FUS. In Figure 3, the affected area of FUS accounted for nearly half of the total iris appearance and was mostly located in the pupillary collar. In contrast, the affected area of non-FUS images (Figure 4) was unevenly distributed, including in the pupil or around the periphery of the iris. The affected areas of our SET-ResNext50 model correspond to those identified by the clinicians for diagnosis. In summary, SET-ResNext50 showed the best level of performance in our study and emphasized the most important clues of the image that pointed to the classification results.

### Comparison With Ophthalmologists

Three resident ophthalmologists and three attending ophthalmologists were included to detect FUS. The average accuracy is 0.709 for attending ophthalmologists from our uveitis center, which is higher than that (0.597) for resident ophthalmologists from other eye institutes. There is significant difference in accuracy between these two groups (*p* = 0.024). Moreover, there was a huge performance gap between ophthalmologists and DCNNs (Table 2). The average accuracy of ophthalmologists is significantly lower than that of DCNNs (*p* < 0.01). As shown in Table 2, comparing the accuracy of ophthalmologists (0.597 and 0.709), the SET-ResNext50 model with the accuracy of 0.930 shows that the latter is superior for detecting FUS.

## Discussion

In this study, we developed DCNNs to prove a well-trained DL method for distinguishing FUS from various anterior uveitis and even identify the diagnostic clues that many clinical ophthalmologists neglect. Our study includes three meaningful conclusions. Firstly, to our knowledge, this is the first initiative to assist ophthalmologists in making a correct diagnosis of FUS using slit-lamp images. Secondly, we trained seven DCNNs and developed a new optimized model (SET-ResNext50). SET-ResNext50 achieved both high accuracy and precision, consistently outperforming other models and the general ophthalmologists. Thirdly, our study provided heat maps that highlighted and showed the location of lesions in slit-lamp images. Applying the DCNNs to assist the detection of hidden lesions can facilitate the clinical diagnosis and treatment process.

Recently, DCNNs have been rapidly popularized in clinical practice to make predictions of diseases automatically. Schlegl et al. (2018) achieved excellent accuracy for the detection and quantification of macular fluid in OCT images by using DL in retinal image analysis. Several studies have suggested that applying a DCNN-based automated assessment of age-related macular degeneration from fundus images can produce results that are similar to human performance levels (Burlina et al., 2017; Grassmann et al., 2018; Russakoff et al., 2019). Intensive efforts to develop automated methods highlight the attraction of these tools for advanced management of clinical disease, especially for diseases like FUS. The slit-lamp microscope is the most widely used auxiliary instrument in clinical practice (Jiang et al., 2018). In busy clinics, taking a mass of slit-lamp images into consideration is inherently impractical and error-prone for ophthalmologists. Therefore, automated DCNNs could be used to screen slit-lamp image data sets, direct the ophthalmologists’ attention to the lesion, and in the near future perform diagnosis independently. Our presented DCNNs with high accuracy for the detection of FUS highlighted the location of lesions and may become widely applicable.

Building and optimizing a new DCNN may require a substantial amount of hyper-parameter tuning time. Therefore, many studies have used classical networks such as Resnet as the backend (Wu et al., 2017). In this study, as the basis of ResNext50, we proposed a mixed “attention” module combining informative attention and spatial attention in our optimized model architecture. We found that SET-ResNext50 with a mixed “attention” module outperformed the models combining with one of the SE and ST modules, indicating that there is mutual promotion between the SE module and ST module. The innovative method of using this mixed module may be useful in other areas of ophthalmology. With too few images or too many training steps, the DL classifier may show overfitting, resulting in the poor generalization of results (Treder et al., 2018). In this study, we used data augmentation to generate new annotated training samples and set an independent test set to evaluate the generalization ability of DCNNs. We found that the performance of our DCNNs was consistently good in the test set, indicating that the models had the generalization ability without overfitting. We compared the effectiveness of DCNNs against ophthalmologists with different experience levels. The performance of attending ophthalmologists was better than that of the resident ophthalmologists, indicating that the misdiagnosis of FUS may be due to a lack of accumulation of clinical experience. As expected, the DCNNs achieving the highest sensitivity while keeping high specificity outperformed the resident and attending ophthalmologists by a large margin. Moreover, our model produced the heat map visualizations to identify the existence of the target areas in images and then generated the output of the classification. In the heat maps, the most apparently affected regions of FUS images were mainly located in the depigmentation of the pupil collar and accounted for nearly half of the overall iris appearance (Figure 3), which generally proved the characteristic of diffuse and uniform iris depigmentation in the vicinity of the pupil in FUS patients. Other uveitis entities like the Posner–Schlossman syndrome may show heterogeneous and uneven iris depigmentation. Iris depigmentation can also be detected in patients with herpetic uveitis, but it usually displays a local appearance. Those signs correspond to the irregular affected areas on the heat maps of non-FUS (Figure 4). Some affected areas in non-FUS cases (like idiopathic chronic anterior uveitis) that were located in the pupil may arise from the presentation of posterior synechiae without iris depigmentation. However, the heat maps produced by DCNNs are challenging and difficult to interpret (Ramanishka et al., 2017). In image-based diagnostic specialties, interpreting the heat map may facilitate a better understanding of the diagnosis.

We realize that our study has several limitations. First, our data of slit-lamp images only included Chinese patients with highly pigmented iris and our findings therefore need to be validated in other ethnic populations. Unfortunately, there is no other public dataset of the FUS patients from different populations to validate our models. Such a dataset would be a significant value for further research and expected to evaluate the performance of other DCNNs in the future. Second, the available data set is relatively small for training or validation. Unlike in other common eye diseases such as age-related macular degeneration or diabetic retinopathy, there are a relatively smaller number of cases with FUS. Third, the program we developed could only distinguish FUS from non-FUS according to the iris change. Further research is expected to combine DCNNs with other clinical findings in the diagnosis of complex diseases. Anyhow, we believe that the method presented here is a meaningful step toward the automated analysis of slit-lamp images and may aid in the detection of FUS.

## Conclusion

In conclusion, we have developed various DCNNs and validated a sensitive automated model (SET-ResNext50) to detect FUS using slit-lamp images. Our presented models achieved both high accuracy and precision, and outperformed general ophthalmologists by a large margin. The SET-ResNext50 model may be the optimal choice to facilitate the diagnosis of FUS. Moreover, the heat map could extract important features from the iris, which proved that DCNNs could be trained to detect specific disease-related changes. The DCNNs are expected to be applied to auxiliary imaging instruments for preliminary screening of diseases, which is of value in future clinical practices.

## Data Availability Statement

The raw data supporting the conclusions of this article will be made available by the authors, without undue reservation.

## Ethics Statement

The studies involving human participants were reviewed and approved by the Ethical Committee of First Affiliated Hospital of Chongqing Medical University (No. 2019356). The patients/participants provided their written informed consent to participate in this study. Written informed consent was obtained from the individual(s) for the publication of any potentially identifiable images or data included in this article.

## Author Contributions

PY, WZ, and ZC: had full access to all of the data in the study and took responsibility for the integrity of the data and the accuracy of the data analysis. WZ, ZC, and HZ: acquisition, analysis, or interpretation of data. RC, LC, and GS: statistical analysis. YZ, QC, CZ, and YW: methodology supervision. WZ and ZC: writing—review and editing. PY: funding acquisition. All authors contributed to the article and approved the submitted version.

## Conflict of Interest

The authors declare that the research was conducted in the absence of any commercial or financial relationships that could be construed as a potential conflict of interest.
